# Susceptibility to febrile malaria is associated with an inflammatory gut microbiome

**DOI:** 10.21203/rs.3.rs-3974068/v1

**Published:** 2024-04-04

**Authors:** Nathan Schmidt, Kristin Van Den Ham, Layne Bower, Shanping Li, Hernan Lorenzi, Safiatou Doumbo, Didier Doumtabe, Kassoum Kayentao, Aissata Ongoiba, Boubacar Traore, Peter Crompton

**Affiliations:** Indiana University School of Medicine; Indiana University School of Medicine; Indiana University School of Medicine; NIAID/NIH; NIAID; University of Sciences, Technique and Technology of Bamako; University of Sciences, Technique and Technology of Bamako; University of Sciences, Technique and Technology of Bamako; University of Sciences, Technique and Technology of Bamako; National Institutes of Health

## Abstract

Malaria is a major public health problem, but many of the factors underlying the pathogenesis of this disease are not well understood. Here, we demonstrate in Malian children that susceptibility to febrile malaria following infection with *Plasmodium falciparum* is associated with the composition of the gut microbiome prior to the malaria season. Gnotobiotic mice colonized with the fecal samples of malaria-susceptible children had a significantly higher parasite burden following *Plasmodium* infection compared to gnotobiotic mice colonized with the fecal samples of malaria-resistant children. The fecal microbiome of the susceptible children was enriched for bacteria associated with inflammation, mucin degradation, gut permeability and inflammatory bowel disorders (e.g., *Ruminococcus gauvreauii, Ruminococcus torques, Dorea formicigenerans, Dorea longicatena, Lachnoclostridium phocaeense* and *Lachnoclostridium* sp. YL32). However, the susceptible children also had a greater abundance of bacteria known to produce anti-inflammatory short-chain fatty acids and those associated with favorable prognosis and remission following dysbiotic intestinal events (e.g., *Anaerobutyricum hallii, Blautia producta* and *Sellimonas intestinalis)*. Metabolomics analysis of the human fecal samples corroborated the existence of inflammatory and recovery-associated features within the gut microbiome of the susceptible children. There was an enrichment of nitric oxide-derived DNA adducts (deoxyinosine and deoxyuridine) and long-chain fatty acids, the absorption of which has been shown to be inhibited by inflamed intestinal epithelial cells, and a decrease in the abundance of mucus phospholipids. Nevertheless, there were also increased levels of pseudouridine and hypoxanthine, which have been shown to be regulated in response to cellular stress and to promote recovery following injury or hypoxia. Overall, these results indicate that the gut microbiome may contribute malaria pathogenesis and suggest that therapies targeting intestinal inflammation could decrease malaria susceptibility.

## INTRODUCTION

*Plasmodium falciparum* infection remains a major cause of morbidity and mortality in tropical and subtropical regions throughout the world. There were 249 million cases and 608,000 deaths due to malaria in 2022, the majority of which occurred among children in the World Health Organization African region^1^. The clinical manifestation of *P. falciparum* infection can range from asymptomatic to severe and fatal symptoms, including respiratory distress and cerebral malaria. The proportion of *P. falciparum* infections that are asymptomatic is known to increase as the transmission intensity and the prevalence of malaria in the community increases^2^. Moreover, individuals residing in areas with moderate-to-high levels of transmission typically develop protection from severe symptoms in early childhood and protection from febrile symptoms by early adolescence^3–5^. However, the factors responsible for modulating malaria pathogenesis have not been fully defined.

The gut microbiome is increasingly recognized as playing a role in the etiology of numerous diseases^6–8^, including those caused by intestinal and extraintestinal pathogens^9–11^. The susceptibility of mice to *Plasmodium* infection, as measured by their parasite burden, has been established to be highly dependent on the composition of their gut microbiome^10,12^, but the influence of the gut microbiome on clinical malaria outcomes in humans is less well characterized. In a cross-sectional study, the composition of the fecal microbiome was shown to be different between Ugandan infants (aged 0.5–4 years) with severe malarial anemia and those with asymptomatic *P. falciparum* infection^12,13^, but whether the differences in the microbiome engendered the differences in malaria severity or were the result of the differential severity is unclear. Additionally, a longitudinal study in a cohort of Malian children and adults (aged 0.25–25 years) found that the fecal microbiome composition before the start of the malaria season correlated with the prospective risk of *P. falciparum* infection, but not with the development of febrile malaria^14^. Since the risk of developing febrile malaria decreases with age/malaria exposure in endemic areas, and the gut microbiome also varies with age^15^, it is possible that the wide age range of participants in this study left it underpowered to detect age-specific correlations between the microbiome and the risk of febrile malaria.

Here, we demonstrate that the fecal microbiome of older Malian children (aged 10 years) before the start of the malaria transmission season correlates with the development of febrile malaria following *P falciparum* infection during the ensuing malaria season. Gnotobiotic mice colonized with fecal samples collected from malaria-susceptible children had a significantly higher parasite burden following *Plasmodium* infection than those colonized with fecal samples from malaria-resistant children. Susceptibility to febrile malaria correlated with a greater abundance of bacteria associated with inflammatory bowel disease (IBD), intestinal mucus barrier degradation, and inflammation, but additionally with short-chain fatty acid (SCFA) production and remission following dysbiotic intestinal events. Metabolomics validated the potential metabolic activity indicated by the metagenomics analysis, with the fecal samples of the susceptible children having increased levels of metabolites associated with nitric oxide-induced inflammatory damage and impaired barrier function, but also those associated with recovery following intestinal dysbiosis. This longitudinal study provides the first demonstration that the gut microbiome plays a role in the clinical outcome of an extraintestinal disease in humans. Further investigation into the intestinal environment of children who are differentially susceptible to febrile malaria and the mechanisms connecting the inflammatory and recovery processes with *Plasmodium* infection-induced pathogenesis has the potential to lead to interventions that limit the severity of malaria symptoms.

## RESULTS

### Classification of Malian children as resistant or susceptible to febrile malaria

The study was conducted in Kalifabougou, Mali, where *P. falciparum* transmission is seasonal; the majority of infections occur between July and December each year^16^. Children aged 6 to 10 years old (n = 181) were enrolled in a prospective cohort study from May 2014 to March 2015. Of the 181 children, 156 were included in the microbiome analysis after removing children who were missing fecal samples (n = 2) or who did not have a detected *P. falciparum* infection (by PCR or microscopy) during the study period (n = 23) (Table 1).

We were interested in determining if the gut microbiome of these children was associated with the clinical outcome of *P. falciparum* infection. Children were initially characterized as ‘susceptible’ if they experienced at least one febrile malaria episode during the study period and as ‘resistant’ if they had no febrile malaria episodes during the study period despite having at least one asymptomatic *P. falciparum* detected by PCR. Asymptomatic *P. falciparum* infections were detected at monthly scheduled visits, while febrile malaria episodes were detected at the same scheduled visits and during unscheduled sick visits. Almost all children under 10 years of age experienced at least one episode of febrile malaria during the study period (83 out of 86 children), whereas a quarter of children aged 10 years (16 out of 70 children) were resistant (infected without symptoms) (Table 1). This finding is consistent with the well-established phenomenon wherein individuals in high-transmission areas gradually develop partial protection from febrile malaria by early adolescence^3–5^; therefore, to minimize the confounding effect of age, we focused the microbiome analysis on children 10 years of age.

### Susceptibility to febrile malaria corresponds with the gut microbiome composition

**Figure 1:**
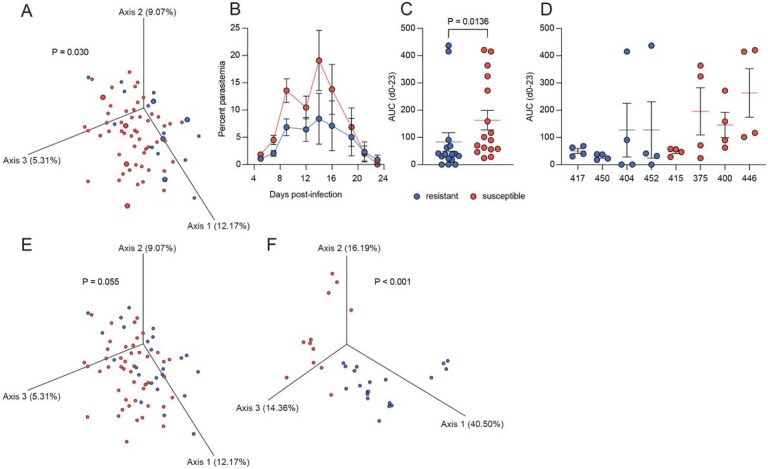
Microbiome composition correlates with susceptibility to febrile malaria in children and high parasite burden in mice. A) Principal coordinate analysis (PCoA) plot of the Bray-Curtis dissimilarity of the human fecal samples. Gavage samples are highlighted by increased point size. B) Parasitemia and C) AUC of the gavaged gnotobiotic mice by resistant and susceptible outcome groups, and D) parasitemia by individual gavage groups. PCoA plots of the Bray-Curtis dissimilarity for the E) human and the F) murine fecal samples using the updated resistant definition. The P-value for the Bray-Curtis distance was determined using PERMANOVA and the P-value for parasitemia was determined using the Mann Whitney U test.

Since transmission of *P. falciparum* in Mali is seasonal, the dry season (January to May) offers an approximate wash-out period between successive transmission seasons. Consequently, we analyzed the fecal samples collected in May to determine if the microbiome composition prior to the start of the transmission season prospectively correlated with the risk of febrile malaria during the ensuing season. 16S rRNA sequencing was performed, and the microbiome composition of the pre-transmission season fecal samples was found to be significantly different between the malaria-resistant and -susceptible groups (P = 0.030) using Bray-Curtis dissimilarity (Fig. 1A).

#### Parasite burden of the humanized gnotobiotic mice corresponds with the susceptibility of the human donor to febrile malaria

A humanized gnotobiotic mouse model was employed to determine if there was a causal link between the gut microbiome of the Malian children and malaria susceptibility. Fecal samples were chosen to cover the space spanned by the principal components (Fig. 1A). Four susceptible children (subject IDs 375, 400, 415, and 446) and four resistant children (subject IDs 404, 417, 450, and 452) were selected and the May fecal sample from each child was used to colonize four sex-matched germ-free mice. One week following the final gavage, the mice were infected with *Plasmodium yoelii* and parasitemia was followed until the infection cleared.

Overall, the mice that were colonized with the fecal samples from the resistant children had significantly lower parasitemia (P = 0.0136) than the mice that were colonized with the fecal samples from the susceptible children (Fig. 1B and C). One mouse colonized with the fecal sample of resistant donor 404 and one mouse colonized with the fecal sample of resistant donor 452 had high parasitemia (Fig. 1D).

True germ-free mice (GF) typically have low levels of parasitemia following infection with *P. yoelii,* but it is relatively common to observe high parasitemia in a small subset of these mice (Supp Fig. 1A and B). Additionally, this phenomenon has been observed in mice that are known to possess a microbiome associated with a low level of parasitemia (Tac)^10^ (Supp Fig. 1 and B).

Mice colonized with the fecal sample of susceptible donor 415 exhibited low parasitemia. Given this outlier, we more closely examined the incidence of asymptomatic *P. falciparum* infections (detected by PCR) and febrile malaria episodes in the four susceptible donors. Over the study period, susceptible subjects 375, 400 and 446 each experienced 3 to 4 febrile malaria episodes while no asymptomatic infections were detected (Table 2), consistent with their malaria-susceptible phenotype. In contrast, subject 415 experienced two febrile malaria episodes and was asymptomatically infected at five timepoints (Table 2), suggesting a higher degree of malaria resistance.

A similar analysis of the cumulative incidence of asymptomatic *P. falciparum* infections and febrile malaria episodes among all 156 children showed that 26 children initially classified as malaria-susceptible had both asymptomatic and febrile infections, seven of which had 1 to 2 febrile malaria episodes and were asymptomatically infected at five or more timepoints during the study period (Table 2). Re-analysis of the 16S rRNA sequencing after re-classifying these seven children as malaria-resistant showed a similar trend to that which was observed using the initial definition of malaria resistance and susceptibility (P = 0.055) (Fig. 1E).

16S rRNA sequencing was performed on the fecal samples collected from the mice on the day of infection and the engrafted microbiomes were determined to be significantly different (P < 0.001) between the groups of mice colonized with fecal samples from children reclassified as malaria-resistant versus malaria-susceptible using Bray-Curtis dissimilarity (Fig. 1F).

### Bacteria increased in the high parasite burden mice are associated with impaired gut barrier function

**Figure 2:**
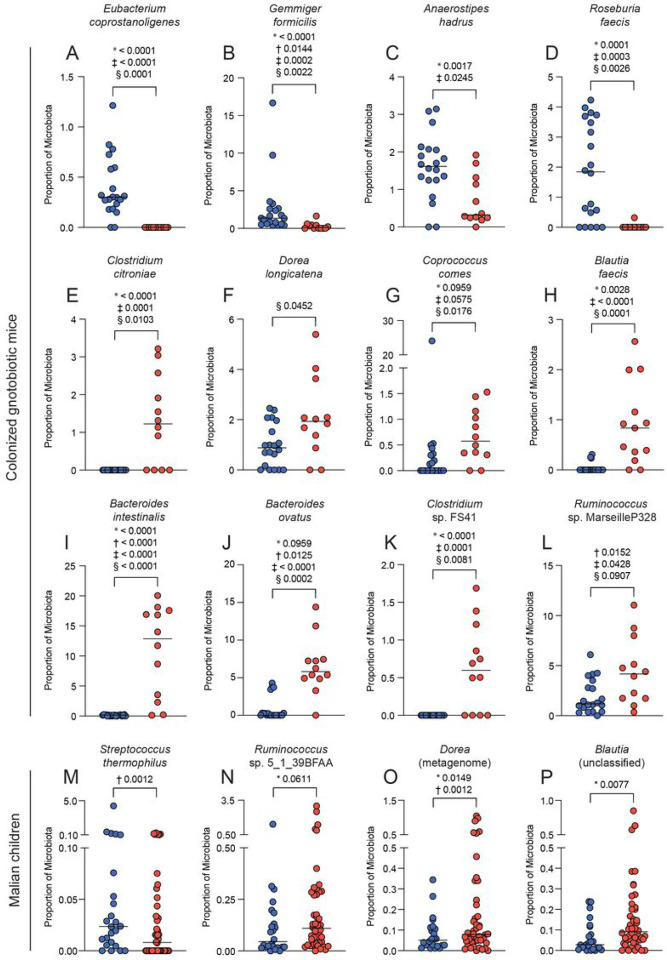
Taxa associated with susceptibility and resistance using 16S rRNA sequencing. Percentage of the mouse microbiome that is A) *Eubacterium coprostanoligenes,* B) *Gemmiger formicilis,* C) *Anaerostipes hadrus,* D) *Roseburia faecis*, E) *Clostridum citroniae*, F) *Dorea longicatena*, G) *Coprococcus comes*, H) *Blautia faecis*, I) *Bacteroides intestinalis*, J) *Bacteroides ovatus*, K) *Clostridium* sp. FS41, and L) *Ruminococcus* sp. Marseille-P328. Percentage of the human microbiome that is M) *Streptococcus thermophilus,* N) *Ruminococcus* sp. 5_1_39BFAA, O) *Dorea* (metagenome), and P) *Blautia* (unclassified). P-values determined using DESeq2 (*), corncob (†), MaAsLin2 (‡) and ALDEx2 (§). Blue circles indicate resistant humans or low parasitemia mice, and red circles indicate susceptible humans or high parasitemia mice.

Differential abundance analysis was performed on the mouse 16S rRNA sequencing data to examine the relationship between the microbiome and parasite burden (Table 3). *Eubacterium coprostanoligenes, Gemmiger formicilis, Anaerostipes hadrus* and *Roseburia faecis* were significantly increased in the low parasitemia mouse groups (Fig. 2A–D). *Clostridium citroniae, Dorea longicatena, Coprococcus comes, Blautia faecis, Bacteroides intestinalis,* and *Bacteroides ovatus,* as well as two unclassified species, *Clostridium* sp. FS41, *Ruminococcus* sp. Marseille-P328, were significantly increased in the high parasitemia mouse groups (Fig. 2E–L). Interestingly, many of the bacteria that were significantly more abundant in the high parasite burden mice have previously been shown to be associated with impaired gut barrier function and to be enriched during IBD^17–19^.

#### Resistance to febrile malaria was associated with Streptococcus thermophilus and susceptibility with Eubacteriales

Differential abundance analysis was performed on the human 16S rRNA sequencing data to further investigate the association of the microbiome with susceptibility (Table 3). *Streptococcus thermophilus* was significantly more abundant in the microbiome of the resistant children (Fig. 2M), and unclassified species of *Ruminococcus, Dorea,* and *Blautia* were significantly more abundant in the susceptible children (Fig. 2N–P).

### Discrimination of malaria-resistant and -susceptible children improved by increased sequencing depth

Since we demonstrated that the microbiome is causally linked to malaria susceptibility using a gnotobiotic mouse model, we performed shotgun metagenomics sequencing on the human samples to better understand the microbiome composition and the interactions underlying susceptibility to febrile malaria through the increased sequencing depth and the improved classification at the species level afforded by this method. The Bray-Curtis dissimilarity was significantly different (P < 0.001) between the resistant and susceptible groups using the metagenomic sequencing data (Supp Fig. 2A).

### The malaria-susceptible network is more interconnected than the resistant network

**Figure 3:**
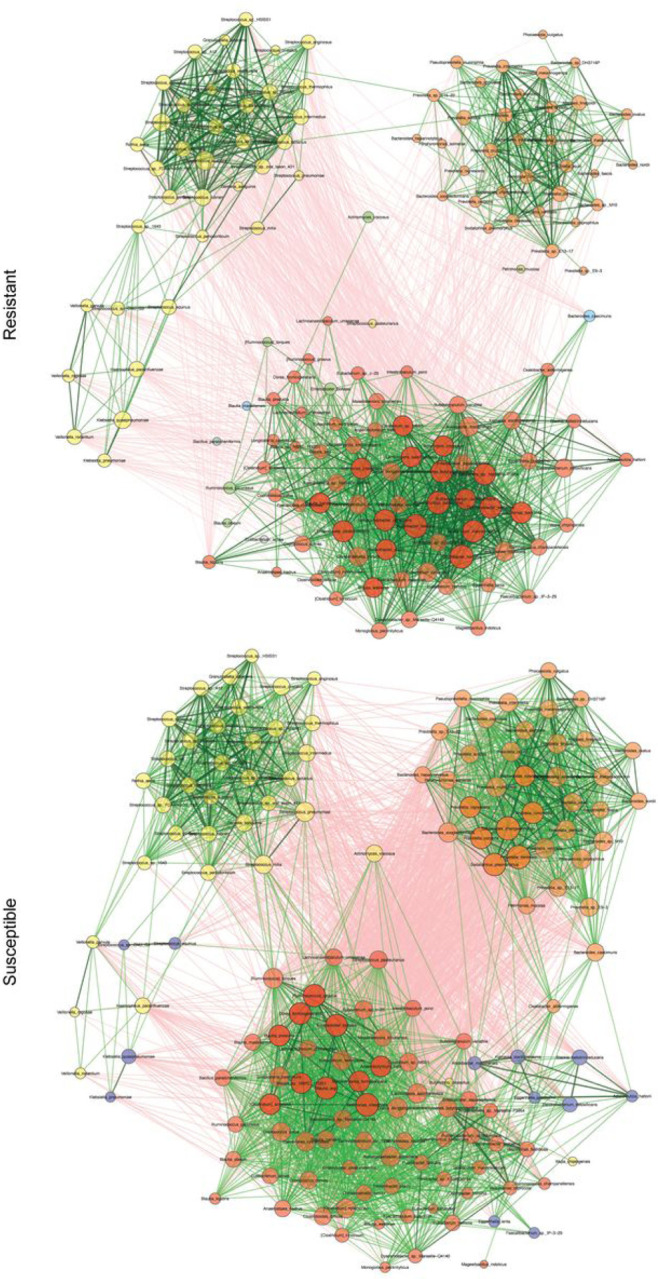
Network of top 100 nodes by degree centrality for resistant and susceptible children. Size of nodes is indicative of degree centrality (number of connected nodes). Hubs (nodes with a degree centrality value above the empirical 95% quantile of all degree centralities in the network) are further highlighted with bold borders and decreased transparency. Nodes are colored by cluster (identified using greedy modularity optimization). Green edges indicate positive interactions, red edges indicate negative interactions, and absolute edge weight is indicated by transparency of the edge.

Network analysis was performed on the samples to determine if there were differential interactions between the bacteria in the resistant and susceptible children. Overall, there were three principal clusters shared by both networks; the first predominated by *Streptococcus* and *Veillonella* species, the second by *Prevotella* and *Bacteroides* species, and the third by species in the order Eubacteriales (Fig. 3; Supp Fig. 3). Additionally, while the edges (connections between two taxa) were in general positive (indicative of positive correlation between the species) and similar between the two networks (71.97% in resistant versus 66.11% in susceptible), the majority of the inter-cluster interactions were negative (indicative of negative correlation between the species; Fig. 3). The edge density (proportion of total possible edges – a measure of network connectivity) of the susceptible network was nearly twice that of the resistant network (0.059 in resistant versus 0.112 in susceptible) (Supp Fig. 4A). This trend was also observed for the *Streptococcus/Veillonella* (0.071 versus 0.111), the *Prevotella/Bacteroides* (0.042 versus 0.149), and the Eubacteriales (0.096 versus 0.133) clusters individually (Supp Fig. 4B-D).

The Jaccard index was significantly different (Jacc = 0.029; P(J ≤ j) = 0.000019) for the two sets of hubs (the most connected taxa in each of the networks), indicating that the two sets of hubs were significantly more different than expected at random; the only shared hub was *Sellimonas intestinalis* in the Eubacteriales cluster (Table 5). Both the resistant and susceptible networks had hubs in the Eubacteriales cluster; the susceptible network also had hubs in the *Prevotella/Bacteroides* cluster, and neither network had hubs in the *Streptococcus/Veillonella* cluster (Table 5). Similar to what was observed for the full network, the edge density for the hubs was lower in the resistant network compared to the susceptible network (0.209 versus 0.277; Table 5).

Interestingly, the edges connected to the hubs in the resistant network had higher absolute median edge weights (a measure of the strength of the correlation between the taxa^20^) compared to the edges connected to the hubs in the susceptible network, for both positive (0.659 versus 0.553) and negative edges (−0.616 versus − 0.464) (Supp Fig. 4E and F). Higher absolute median edge weights were also observed for the total resistant network compared to the total susceptible network (Supp Fig. 4G and H), for both the positive (0.656 versus 0.512) and negative edges (−0.611 versus − 0.443).

#### Eubacteriales cluster is associated with malaria-susceptible children and Prevotella/Bacteroides and Streptococcus/Veillonella clusters are associated with resistant children

**Figure 4:**
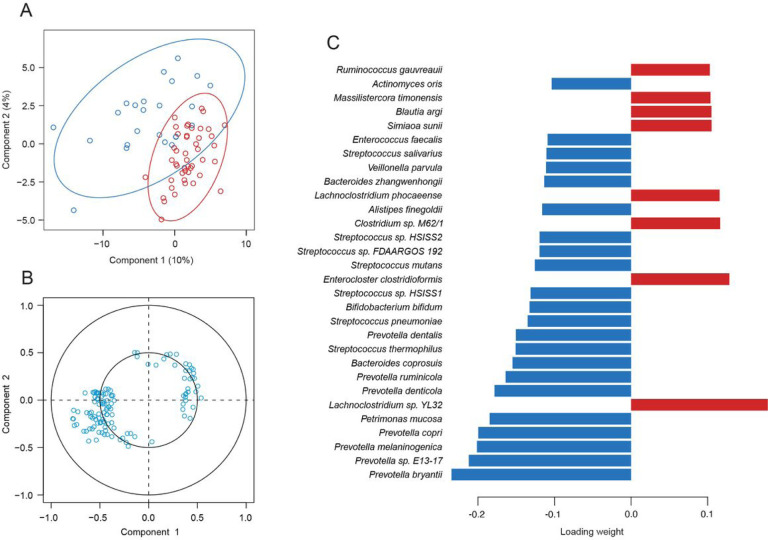
Species from the *Prevotella/Bacteroides* and *Streptococcus/Veillonella* clusters correlate with the resistant children and species from the Eubacteriales cluster correlate with the susceptible children. A) Sample plot, B) correlation circle plot, and C) loadings plot for the sPLS-DA of the metagenomics samples (the top 30 taxa by loading weight are listed and the colors indicate in which group the taxa has the maximum median count). The ellipses represent 95% confidence intervals in the sample plot and a correlation coefficient cut-off of 0.35 was used for the correlation circle plot.

Sparse partial least squares discriminant analysis (sPLS-DA) was performed on the metagenomics sequencing samples in order to better understand the correlation of the bacteria with each other and with susceptibility to febrile malaria. The samples were projected onto the space spanned by the first two components (Fig. 4A), which demonstrated moderate separation of the children by malaria outcome along the first component. One group of bacteria was positively correlated with component one and one group of bacteria was negatively correlated with component one (Fig. 4B), approximately corresponding to the resistant and susceptible groups on the sample plot.

Closer examination of the correlation circle plot permitted a more granular characterization of the relationships between the different bacteria. The bacteria that correlated with the resistant group of children were those that were largely found within the *Prevotella/Bacteroides* and the *Streptococcus/Veillonella* clusters in the network analysis (Supp Fig. 5A). Conversely, the majority of the bacteria that correlated with the susceptible group were those that were detected in the Eubacteriales cluster (Supp Fig. 5B). In agreement with the correlation circle plot, the bacteria with the top loading weights (a measure of how much they contribute to the component) that had a higher median abundance in the resistant group were found primarily within the *Prevotella/Bacteroides* and the *Streptococcus/Veillonella* clusters, and the bacteria that had a higher median abundance in the susceptible group were found in the Eubacteriales cluster (Fig. 4C).

### Malaria susceptibility associated with mucolytic bacteria and butyrate-producing probiotics

**Figure 5:**
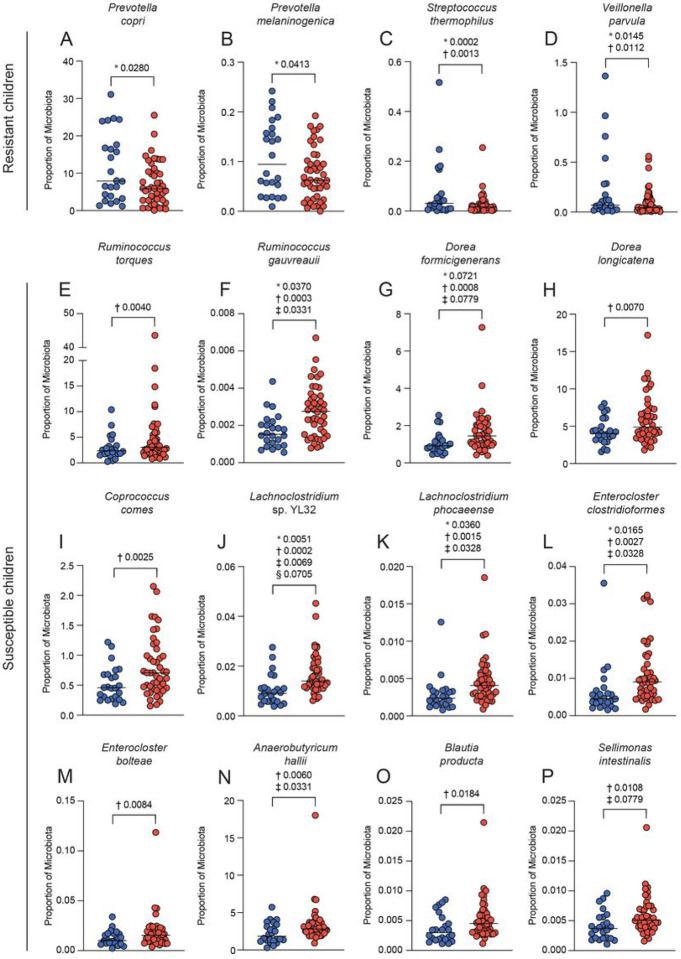
Taxa associated with susceptibility and resistance in the children using shotgun metagenomics. Percentage of the microbiome that is A) *Prevotella copri,* B) *Prevotella melaninogenica,* C) *Streptococcus thermophilus,* D) *Veillonella parvula,* E) *Ruminococcus torques,* F) *Ruminococcus gauvreauii,* G) *Dorea formicigenerans,* H) *Dorea longicatena,* I) *Coprococcus comes,* J) *Lachnoclostridium* sp. YL32, K) *Lachnoclostridium phocaeense,* L) *Enterocloster clostridioformis,* M) *Enterocloster bolteae,* N) *Anaerobutyricum hallii,* O) *Blautia producta,* and P) *Sellimonas intestinalis*. P-values determined using DESeq2 (*), corncob (†), MaAsLin2 (‡) and ALDEx2 (§). Blue circles indicate resistant humans and red circles indicate susceptible humans.

Differential abundance analysis was performed on the samples to determine if the disparate interaction networks within the resistant and susceptible microbiomes were related to differential prevalence of the bacteria (Table 6). *Prevotella copri, Prevotella melaninogenica, S. thermophilus, Veillonella parvula, Prevotella bryantii, Prevotella* sp. E13–17, *Petrimonas mucosa,* and *Streptococcus pneumoniae* were significantly increased in the resistant children (Fig. 5A–D; Supp Fig. 6A-D). *Ruminococcus torques, Ruminococcus gauvreauii, Dorea formicigenerans, D. longicatena, C. comes, Lachnoclostridium* sp. YL32, *Lachnoclostridium phocaeense, Enterocloster clostridioformis, Enterocloster bolteae, Anaerobutyricum hallii, Blautia producta, S. intestinalis, Clostridium* sp. M62/1, *Clostridium scindens, Marvinbryantia formatexigens, Massilistercora timonensis, Blautia argi, Blautia liquoris, Blautia hansenii,* and *Butyrivibrio crossotus* were more abundant in the susceptible children (Fig. 5E–P and Supp Fig. 6E-L).

*Bacteroides* were recently demonstrated to be significantly increased in Ugandan infants with severe malaria anemia compared to infants with asymptomatic *P. falciparum* infection^12^. *Bacteroides caccae, Bacteroides cellulosilyticus, Bacteroides fragilis* and *Bacteroides uniformis were* significantly increased in the susceptible Malian children (Table 6), although this appears to be largely the result of outliers (Supp Fig. 7A-D).

Similar to the high parasite burden mice, the susceptible children had increased abundances of bacteria that have been associated with mucin degradation, impaired gut barrier function and IBD (e.g., *R. gauvreauii, R. torques, D. formicigenerans, D. longicatena, L. phocaeense* and *L*. sp. YL32)^17–19,21–23^. However, several bacteria that are known SCFA-producers and that have been associated with intestinal homeostasis recovery following dysbiotic events were also significantly more abundant in the susceptible children (e.g., *A. hallii, B. producta* and *S. intestinalis)*^24–31^.

### Susceptibility associated with metabolites indicative of inflammatory gut barrier damage

**Figure 6:**
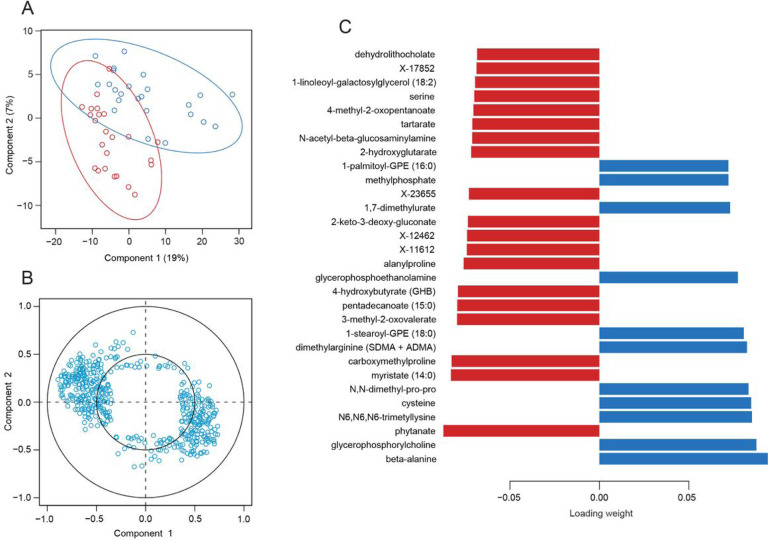
Metabolite loading weights are evenly distributed. A) Sample plot, B) correlation circle plot, and C) loadings plot for the sPLS-DA of the metabolomics samples (the top 30 metabolites by loading weight are listed and the colors indicate in which group the metabolite has the maximum median count). The ellipses represent 95% confidence intervals in the sample plot and a correlation coefficient cut-off of 0.35 was used for the correlation circle plot.

Since the susceptible children had significantly increased abundances of bacteria that have previously been associated with inflammation and mucin degradation, untargeted metabolomics was performed to determine if the metabolic activity of the microbiota within the Malian children supported the association of impaired gut barrier function with susceptibility to febrile malaria. Moderate separation of the resistant and susceptible children was observed along the first component when the samples were projected onto the space spanned by the first two components (Fig. 6A), and two groups of metabolites were observed to approximately correspond with the resistant and susceptible groups of children (Fig. 6B). The loading weights of the top thirty metabolites for component one were relatively evenly distributed (Fig. 6C), suggesting that the differences in metabolic activity may have been the result of comprehensive changes in pathways, rather than inordinate changes in a few select metabolites. Thus, the range of the metabolites of interest was extended to include all metabolites with an absolute loading weight of at least 0.05 (Table 7), which brought several metabolite categories into relief.

Fifteen metabolites associated with glycerophospholipid metabolism had a higher median count in the resistant children, including sarcosine, betaine, trimethylamine-N-oxide (TMAO), trimethyllysine, stearoylcarnitine, and 9-palmitic acid hydroxystearic acid (PAHSA)/12-PAHSA, and nine phospholipids: glycerophosphorylcholine (GPC), three lysophosphatidylcholines (LPC) (1-oleoyl-GPC, 1-stearoyl-GPC, and 1-palmitoyl-GPC), glycerophosphorylethanolamine (GPE), and four lysophosphatidylethanolamines (LPE), (1-margaroyl-GPE, 1-oleoyl-GPE, 1-stearoyl-GPE, and 1-palmitoyl-GPE) (Supp Fig. 8A; Supp Fig. 9A-D). Interestingly, putrescine was also shown to have a higher median level in the resistant children (Table 7), and *V. parvula,* which is more abundant in the resistant children (Fig. 5D), requires putrescine for growth, as it is incorporated into its peptidoglycan^32^.

Eleven metabolites associated with purine and pyrimidine metabolism had higher median counts in the susceptible children: deoxyinosine, deoxyuridine, deoxycytidine, N6-methyladenosine (m6A), 5’- methyluridine (m5U), pseudouridine, xanthine, hypoxanthine, thymidine, thymine and orotate (Supp Fig. 8B; Supp Fig. 9E-H). In addition to the metabolites associated with nucleotide metabolism, five long chain fatty acids (LCFA) (myristoleate, 10-nonadecenoate, myristate, margarate and pentadecanoate), three sterols (lanosterol, 4-cholesten-3-one and coprostanol), and eighteen unknown metabolites (Table 7; Supp Fig. 9I-P) were correlated with the susceptible children. Many of the unknown metabolites grouped together on the correlation circle plot, indicating a high level of positive correlation and potentially a shared metabolic pathway (Supp Fig. 10).

Consistent with the potential metabolic activity suggested by the metagenomics data, the metabolomics data indicated the presence of nitrosative stress and impaired gut barrier function (e.g., deoxyinosine and deoxyuridine) within the susceptible children^33–36^, but additionally supported the presence of a dynamically regulated anti-inflammatory recovery component (e.g., hypoxanthine and pseudouridine)^37–39^.

## DISCUSSION

Malaria remains a major cause of morbidity and mortality in low- and middle-income countries. Individuals living in areas with moderate-to-high *P. falciparum* transmission typically develop protection from febrile symptoms by early adolescence, but the factors underlying this transition are not well understood. We observed in a cohort of children aged 6 to 10 years old, that children younger than 10 years of age were generally susceptible to febrile malaria following *P. falciparum* infection, while a quarter of the 10-year-old children were resistant. The microbiome of the 10-year-old children in May (before the start of the transmission season) was significantly different between children that were malaria-resistant or -susceptible during the subsequent transmission season. Furthermore, gnotobiotic mice colonized with the pre-transmission season fecal sample of susceptible children had a significantly higher parasite burden following infection with *P. yoelii* compared to gnotobiotic mice colonized with the fecal sample of resistant children.

Network analysis of the human fecal metagenomics samples revealed three principal clusters shared by both the resistant and the susceptible microbiomes: the first predominated by *Streptococcus* and *Veillonella* species, the second by *Prevotella* and *Bacteroides* species, and the third by species in the order Eubacteriales. The species within the susceptible microbiome were more interconnected than those within the resistant microbiome (higher edge density); however, the average strength of the individual interactions was greater in the resistant network (higher edge weights). The lower number of relatively stronger correlations between the bacteria in the resistant network is potentially indicative of metabolic interactions that are more specific and tightly controlled, while the high number of low strength correlations in the susceptible network may be suggestive of the bacteria responding in an overall similar manner to the environment within the colon. The sPLS-DA and the differential abundance analysis of the whole genome shotgun metagenomics data found that the majority of the bacteria that were associated with/significantly more abundant in the resistant children were from the genera *Prevotella, Streptococcus* and *Veillonella,* while the majority of the bacteria that were associated with/significantly more abundant in the susceptible children were from the order Eubacteriales.

*P. copri,* which was more abundant in the resistant children using shotgun metagenomics, has been associated with contradictory impacts on human health, likely due to the high genetic diversity present in this species^40–42^. The different strains within the *P. copri* complex have been linked to country of origin and fiber intake, and are associated with substantial functional diversity^41,42^. Higher fiber levels correlated with increased potential for the degradation of complex carbohydrates by this species, whereas omnivore diets were linked to an increased incidence of metabolic syndrome in individuals with higher levels of *P. copri*^43,44^. Given the high fiber content in the diet of these children^45^, it seems likely that the increased abundance of *P. copri* in the resistant children is associated with improved degradation of complex carbohydrates. Several *Prevotella-associated* operational taxonomic units (OTUs) were also found to be differentially abundant in the human 16S rRNA sequencing data, but it is difficult to make direct comparisons with the shotgun metagenomics, as the human 16S rRNA data was classified using the SILVA database, which divides *Prevotellaceae* into multiple non-monophyletic groups, each of which are associated with several individual OTUs.

**Figure 7:**
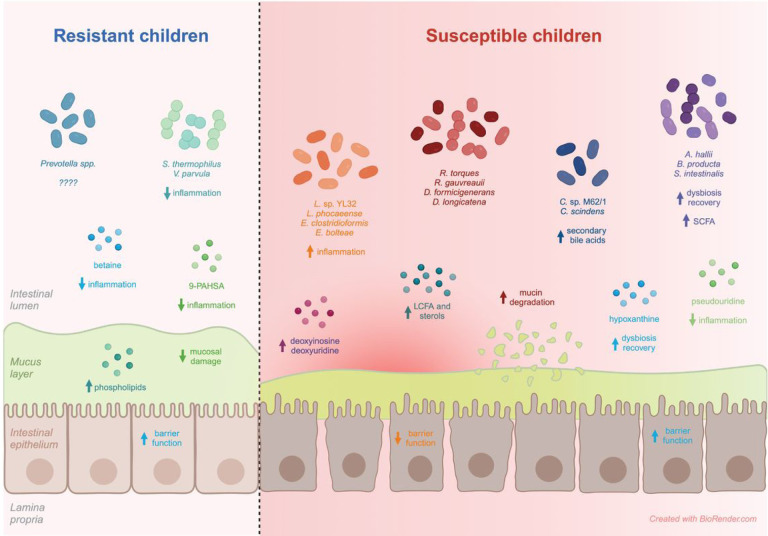
Features associated with inflammation and impaired gut barrier function are enriched in children susceptible to febrile malaria symptoms. Created with BioRender.com.

*S. thermophilus,* which was found to be significantly increased in the resistant children by both shotgun metagenomics and 16S rRNA sequencing, has been shown to be capable of reducing inflammation in a murine model of sepsis^46^. Moreover, *V. parvula,* which was increased in the resistant children, has been shown to modulate the immune response in *in vitro* co-stimulation experiments with different strains of *Streptococcus*^47^. Thus, the increased abundance of *S. thermophilus* and *V. parvula* in the resistant children may have contributed to a more anti-inflammatory intestinal environment (Fig. 7).

The species associated with low parasite burden in the mice were different than those associated with resistance to febrile malaria in children. *G. formicilis, A. hadrus* and *R. faecis,* which were increased in the low parasite burden mice, are known producers of SCFA, which promote gut barrier integrity and reduce inflammation^48–50^. Furthermore, G. *formicilis, A. hadrus,* and *R. faecis* have been shown to be increased in healthy controls compared to ulcerative colitis (UC) and Crohn’s disease (CD), irritable bowel syndrome (IBS), and CD, respectively^49–51^. Moreover, *E. coprostanoligenes,* which was also significantly more abundant in the low parasite burden mice, was shown to be enriched by oroxylin A treatment in conjunction with improved protection of the colonic mucus barrier and alleviation of colitis in mice^52^.

Many of the bacteria that were more abundant in the susceptible children were associated with inflammation, impaired gut barrier function, and IBD (Fig. 7). *R. torques, R. gauvreauii, D. formicigenerans* and *D. longicatena* are mucolytic bacteria; they possess glycoside hydrolases that allow them to initiate mucin degradation by releasing the sialic acids from the non-reducing ends of glycans, impairing gut barrier function^17,18^. Accordingly, increased abundance of these bacteria has been associated with IBD^19,21–23^. *C. comes,* while not in possession of glycoside hydrolases, has also been shown to grow with mucin as the main carbon source^53^ and to be enriched during IBD^19^. *L*. sp. YL32 was positively correlated with gut permeability and inflammation^54^ and L. *phocaeense* was shown to be enriched in patients with active IBD^55^. Finally, *E. clostridioformis* and *E. bolteae* were both shown to be significantly enriched in CD patients^51,56^.

Interestingly, the susceptible children also had increased abundance of several bacteria that have been shown to correspond with favorable prognosis and remission following intestinal dysbiosis (Fig. 7). *A. hallii* cannot degrade complex oligo- and polysaccharides, but participates downstream in mucin cross-feeding, and produces SCFA from mucin-derived monosaccharides^24,25^. While *A. hallii* has been shown to be enriched in IBD groups compared to control groups^19^, patients who achieved remission after fecal microbiota transplantation (FMT) treatment of UC had increased abundance of A. *hallii* and SCFA^26^, potentially suggesting that the presence of *A. hallii* during IBD is indicative of a favorable prognosis. An increase in SCFA was also associated with the amelioration of dextran sulfate sodium (DSS)-induced colitis in mice following oral administration of *B. producta*^27^, and *S. intestinalis* has been shown to be increased in patients during homeostasis recovery following dysbiosis events^28–31^.

Achievement of remission after FMT treatment of UC has also been associated with increased levels of secondary bile acids^26^, and treatment with secondary bile acids has been shown to decrease intestinal inflammation in three models of murine colitis^57^. *C*. sp. M62/1 was shown to play a significant role in bile acid metabolism using *in silico* metabolic modelling^58^, and *C. scindens,* which possesses 7α-dehydroxylases that transform primary bile acids into secondary bile acids^59^, was shown to enhance resistance to *Clostridum difficile* infection in a secondary bile acid-dependent manner^60^.

*D. longicatena* and *C. comes* were the only species associated with susceptibility in the Malian children that were also associated with high parasite burden in the colonized mice. However, despite the taxonomical differences, the bacteria that were more abundant in the high parasite burden mice were also associated with impaired barrier function, suggesting that the high parasite burden phenotype engendered in the colonized mice may have been the product of similar metabolic activities to that which was associated with susceptibility in the children, but with a mouse specific microbiome composition. Gavage with *B. intestinalis* following antibiotic depletion of the gut microbiota has been shown to increase ileal damage compared to mice allowed to naturally repopulate their gut microbiota^61^, and *C. citroniae* possesses D-cysteine desulfhydrases, which increase the concentration of colonic sulfides, potentially inhibiting the utilization of butyrate by intestinal epithelial cells^62^. Additionally, similar to the susceptible children, the high parasite burden mice were also associated with an anti-inflammatory component: *B. ovatus* has been shown to reduce mucosal inflammation during DSS-induced colitis^63,64^ and *B. faecis* has been shown to be reduced in patients with CD and to produce SCFA^50^.

Congruent with the potential metabolic activity indicated by the metagenomics data, many of the metabolites associated with the susceptible children were indicative of an inflammatory environment and gut barrier damage (Fig. 7). Deoxyinosine and deoxyuridine are DNA adducts formed through nitrosative deamination that occurs due to the generation of nitric oxide during chronic inflammation^33^ and are thus potential biomarkers of inflammatory processes. m6A is a post-transcriptional RNA modification that is influenced by the microbiota^65^ and has been shown to be involved in the initiation and pathogenesis of IBD^66^. Moreover, the increased levels of LCFAs and sterols in the susceptible children further support an inflammatory intestinal environment and impaired gut barrier function, as cats with chronic inflammatory digestive disorders were shown to have higher levels of LCFAs and animal-derived sterols in their feces compared to healthy cats^34^, and LPS-induced inflammation was shown to inhibit the absorption of LCFA by intestinal epithelial cells, concomitant with an increase in m6A modification levels^35^.

Nevertheless, similar to what was suggested by the metagenomics data, the metabolomics data also supported the presence of a dysbiosis recovery component in the susceptible children (Fig. 7). Pseudouridine modification has been shown to be dynamically regulated in response to cellular stress and artificial pseudouridylation was shown to reduce immune stimulation *in vitro*^37^. Additionally, hypoxanthine was shown to be lower in the fecal samples of IBD patients compared to healthy controls^38^ and to promote intestinal barrier function and recovery following injury or hypoxia^39^. These results suggest that while the intestinal environment in the susceptible children may be in a more inflammatory state than that of the resistant children, there are supplementary mechanisms in place that are potentially preventing excessive damage and/or allowing recovery.

Furthermore, the metabolites associated with the resistant children also agreed with the potential role of impaired gut barrier function in susceptibility to febrile malaria (Fig. 7). Mucus phospholipids play a role in maintaining the intestinal mucus barrier^36^, thus the increased amounts of phospholipids in the resistant children may indicate a more impregnable mucosal barrier. GPC and LPE were shown to be decreased in UC and CD patients^21,67^ and LPC was decreased in UC patients^36,68^. Furthermore, betaine treatment has been shown to attenuate inflammation and upregulate tight junction proteins during DSS-induced colitis^69^, and 9-PAHSA was shown to attenuate the immune response and prevent mucosal damage during DSS-induced colitis^70^.

Interestingly, elevated levels of intestinal damage biomarkers were observed in young children with severe malaria compared to healthy community controls in a study in Uganda^71^, suggesting that inflammatory gut barrier damage may play a general role in worsening the immune response to *Plasmodium* infection and consequently increasing the severity of the infection. However, as was mentioned, the microbiome is known to vary greatly with age^15^, as is protection from malaria symptoms. Thus, if the microbiome is playing a role in the increased intestinal damage observed in the Ugandan infants with severe malaria, it is possible that the specific bacteria and metabolic activity involved are not the same.

Overall, this study demonstrated that the microbiome plays a role in the susceptibility to febrile malaria. Bacteria and metabolites associated with increased inflammation and gut barrier impairment were enriched within the gut microbiome of the susceptible children; however, the metagenomics and metabolomic data also indicated that the microbiome of the susceptible children also possessed features associated with recovery from dysbiosis. It is possible that the inflammatory intestinal environment within the susceptible children is priming the immune response in a manner that renders them more susceptible to the development of febrile symptoms following *P. falciparum* infection. Further research into the dynamics of the differential bacteria and metabolites during febrile *Plasmodium* infection and recovery, including targeted approaches to examine gut barrier function, and into the mechanisms through which these differences influence the pathogenesis of *Plasmodium* infection, has the potential to lead to treatments capable of mitigating malaria severity.

## METHODS

### Study design, participants, and detection of P. falciparum infection

181 children aged 6 to 10 years old were enrolled in a prospective cohort study from May 2014 to March 2015 conducted in Kalifabougou, Mali. A detailed description of this cohort has been previously published^72^. The Ethics Committee of the Faculty of Medicine, Pharmacy and Dentistry at the University of Sciences, Techniques and Technology of Bamako and the Institutional Review Board of the National Institute of Allergy and Infectious Disease, National Institutes of Health approved this study (ClinicalTrials.gov identifier: NCT01322581). Written, informed consent was obtained from the parents and/or guardians of participating children. Two children were removed from this study due to missing fecal samples. To detect asymptomatic *P. falciparum* infections, fingerprick blood samples were collected at monthly scheduled visits and PCR was performed on the dried blood spots as described previously^73^. Positive *P. falciparum* PCRs were considered to represent an asymptomatic infection if there were no reported febrile symptoms for at least 3 weeks following the PCR. Children were considered to have had a febrile malaria infection if they presented to the clinic with fever and *P. falciparum* parasites were detected by microscopic examination of blood smears. Twenty-three children were excluded from further analysis because they had no *P. falciparum* infections detected during the study period by microscopy or by PCR, leaving a total of 156 children for microbiome analysis (Table 1). Aliquots of stool collected during the prospective cohort study were cryopreserved at −80°C in Mali and shipped to the U.S. on dry ice for analysis.

### Mouse husbandry and gnotobiotic experiment

Germ-free, female and male C57BL/6N mice (5–8 weeks old) were purchased from Charles River Laboratories. Mice were housed in autoclaved Tecniplast IsoCage P cages (Tecniplast Group) with ALPHA-dri bedding (Shepherd Specialty Papers, Inc) and Bed-r’Nest nesting material (The Andersons Plant Nutrient Group) under a strict 12 hr light cycle. Cages were changed once every two weeks. The mice were provided *ad libitum* with autoclaved reverse osmosis water and autoclaved 7013 (NIH-31 Modified Open Formula Mouse/Rat Sterilizable Diet) purchased from Inotiv/Envigo. Cages were submerged in a tank of Exspor for 5 min before being opened within an Exspor-sterilized biosafety cabinet (40 min contact time). Additionally, all items used with the mice *(e.g.,* gavage needles) were autoclaved and their packaging wiped down with Exspor before being transferred into the biosafety cabinet. Fecal samples were collected upon arrival and from the sentinels at the end of the experiment and sterility verified by IDEXX BioAnalytics through generic bacteria 16S rRNA gene PCR, and fungal, and aerobic and anaerobic bacteria culture (case numbers 120118–2022 and 125378–2022). All animal experiments were carried out at Indiana University adhering to the local and national regulation of laboratory animal welfare, and all procedures were reviewed and approved by the Indiana University Institutional Animal Care and Use Committees (protocol numbers 19024 and 22010).

May stool samples for the participants with the subject identification numbers 375, 400, 404, 415, 417, 446, 450 and 452 were selected to colonize the gnotobiotic mice. A portion of each fecal sample was scraped off while on dry ice, and diluted in sterile saline at 1:10 (w/v). The fecal suspension was vortexed, and the larger particles allowed to settle on ice, before the supernatant was collected. 200 μL of each fecal suspension was gavaged into four mice each at weekly intervals for four weeks, as was previously described^45^. Fecal samples were collected from the colonized mice and were flash frozen in liquid nitrogen and stored at −80°C.

Mice were infected with *P. yoelii* 17XNL by intraperitoneal injection of 1.5×10^5^ parasitized red blood cells prepared from fresh donor mouse blood. Tail vein blood was collected at the indicated time points to monitor parasitemia (Fig. 1E). The blood was stained with CD45.2-APC (clone 104; BioLegend), TER-119-APC/Cy7 (clone TER-119; BioLegend), dihydroethidium (MilliporeSigma), and Hoechst 34580 (MilliporeSigma) and parasitemia evaluated using flow cytometry on the Attune Next (Invitrogen). Samples were analyzed with FlowJo (Tree Star), and parasitized red blood cells were defined as CD45.2^−^ TER-119^+^ dihydroethidium^+^ Hoechst^+^ cells. The statistical significance of the difference in the area under the curve (AUC) for the parasitemia was determined using the Mann Whitney U test in GraphPad Prism Version 9.4.1.

### DNA sequencing and feature table construction

DNA isolation and sequencing for the human 16S rRNA sequence data was performed by the J. Craig Venter Institute (JCVI). The V4 region of the 16S rRNA gene was amplified using the 515F (GTGCCAGCMGCCGCGGTAA) and 806R (GGACTACHVGGGTWTCTAAT) primer pair and sequenced using an Illumina MiSeq. An in-house pipeline was used by JCVI to construct the feature table, using the SILVA SSU Ref NR99 database (v123) for taxonomic classification. For the mouse 16S rRNA sequencing and the human whole genome shotgun metagenomics sequencing, DNA was extracted from the feces using the QIAamp PowerFecal DNA kit (QIAGEN, Germantown, MD) according to the manufacturer’s instructions. For the mouse 16S rRNA sequencing, the DNA samples were shipped overnight on ice packs to the Genome Technology Access Center (GTAC; Washington University, St. Louis, MO) for 16S rRNA gene sequencing using Multiple 16S Variable Region Species-level Identification (MVRSION), an approach that sequences all 9 hypervariable regions of the 16S rRNA gene with 14 primer pairs^74^. The OTU table was constructed by GTAC and imported into QIIME2^75^. For the human whole genome shotgun metagenomics sequencing, the DNA library preparation and sequencing was performed by the Center for Medical Genomics (CMG) at the Indiana University School of Medicine using the Nextera XT DNA Library Preparation Kit (Illumina) and the NovaSeq 6000 with 150bp paired-end sequencing. Quality control and host sequence removal was performed using KneadData (v0.12.0)^76^. Briefly, FastQC (v0.11.9)^77^ removed overrepresented sequences (> 0.1*%* frequency), Trimmomatic (v0.33)^78^ removed low quality reads and adapters (SLIDINGWINDOW:4:20 MINLEN: 50), TRF (v4.09.1)^79^ removed tandem repeats, and bowtie2 (v2.5.1)^80^ mapped the samples to the human genome assembly GRCh37 (hg37) to remove possible human read contamination. The feature table was created using Kraken2 (v2.1.2)^81^ and Bracken (v2.8.0)^82^ with the pre-built standard Kraken2 database (version k2_standard_20230314). Minimum hit groups was increased to 4 and the confidence score was increased to 0.10.

### Metabolomics

The fecal samples were shipped overnight to Metabolon on dry ice and maintained at −80°C at Metabolon until processing. Briefly, samples were prepared using the automated MicroLab STAR^®^ system from Hamilton Company. Proteins were precipitated with methanol and vigorous shaking for 2 min (Glen Mills GenoGrinder 2000) followed be centrifugation. The samples were then divided into multiple fractions: two fractions for analysis by two separate reverse phase/UPLC-MS/MS methods with positive ion mode electrospray ionization, one fraction for analysis by reverse phase/UPLC-MS/MS with negative ion mode electrospray ionization, and one fraction for analysis by HILIC/UPLC-MS/MS with negative ion mode ESI. Organic solvent was removed using a TurboVap^®^ (Zymark) and the samples stored under nitrogen until analysis. The dried samples were reconstituted in different solvents according to the four methods. Each of the four methods used a Waters ACQUITY ultra-performance liquid chromatography (UPLC) and a Thermo Scientific Q-Exactive high resolution/accurate mass spectrometer interfaced with a heated electrospray ionization (HESI-II) source and Orbitrap mass analyzer operated at 35,000 mass resolution. Two aliquots were analyzed using acidic positive ion conditions, one chromatographically optimized for more hydrophilic compounds (PosEarly) and the other chromatographically optimized for more hydrophobic compounds (PosLate). The third aliquot was analyzed using basic negative ion optimized conditions (Neg), and the fourth aliquot was analyzed via negative ionization following elution from a HILIC column (HILIC). The MS analysis alternated between MS and data-dependent MS^n^ scans using dynamic exclusion. The scan range varied slightly between methods but covered 70–1000 m/z. Raw data was extracted, peak-identified and quality control processed using a combination of Metabolon developed software services.

### Beta diversity analysis

Cumulative sum scaling^83^ was used to normalize the mouse and human 16S rRNA sequencing feature tables, and relative log expression^84^ was used to normalize the human whole genome metagenomics sequencing feature table. Bray-Curtis diversity was calculated using the default settings in QIIME2 (v2022.11.1)^75^ without rarefaction.

### Differential abundance analysis

Mouse and human 16S rRNA sequencing and human whole genome metagenomics sequencing feature tables underwent differential abundance analysis using DESeq2^84^, Corncob^85^, MaAsLin2^86^, and ALDEx2^87^. Feature tables were normalized using the default method for each tool: relative log expression (RLE), no normalization, total sum scaling (TSS), and no normalization, respectively.

### Network analysis

Network analysis was performed on the human whole genome metagenomics sequencing feature table using the NetCoMi package^20^ (default settings were used). Associations between taxa were computed using SparCC^88^. SparCC estimates the correlations between the taxa from log-ratio-transformed variances via an approximation based on the assumption that the underlying network is sparse (taxa are uncorrelated on average). Zeros are handled through a Bayesian approach (observations replaced by random sampling), and nested iterations are used to reinforce the sparsity assumption and to account for the uncertainty due to the random sampling. The calculated associations were sparsified (t-test) and transformed into similarities that were used to build the association networks. Hubs were determined by degree centrality (number of connected nodes) and clusters (densely connected nodes that are sparsely connected to other clusters) were determined using fast greedy modularity optimization^89^ provided by the igraph package^90^.

### sPLS-DA

We applied sPLS-DA (Sparse Partial Least Square - Discriminant Analysis)^91,92^ to select taxa and/or metabolites that are important for discriminating resistant and susceptible microbiomes. We note here our purpose is to discover microbial related features that are important for understanding the phenotype differences, rather than building a classification tool for phenotype prediction. We chose sPLS-DA over other more recent ML/AI tools for this purpose, due to the simplicity of sPLS-DA and that it provides good interpretability for the results.

sPLS-DA assumes that the majority of features are uninformative in the characterization of the different groups, thus a tuning step is performed to select a smaller subset of features^92^. The classification error rate (balanced error rate (BER)) was estimated with respect to the number of features selected (a sequence equal to c(1:10, seq(15, 350, 5)) for the metagenomics analysis and a sequence equal to c(1:10, seq(15, 790, 15)) for the metabolomics analysis), using 12-fold (metagenomics) and 8-fold cross-validation (metabolomics) repeated 50 times for the first five components. The minimum classification error rate was achieved with one component and 160 taxa for the metagenomics analysis and with one component and 765 metabolites for the metabolomics analysis, assessed with a one-sided t-test. Two components were used for visualization purposes. The human whole genome metagenomics sequencing feature table was normalized using RLE^84^ and MetaboAnalystR^93^ was used to normalize and transform the metabolomics feature table (normalization by median and log10 transformation).

## Data Availability

All feature tables and analysis scripts produced as part of this study can be found in the GitHub repository: https://github.com/kmvanden/microbiome_malaria_Mali. The datasets generated and analyzed during the current study are available in the NCBI Sequence Read Archive: to be uploaded upon manuscript acceptance.
